# Evidence for the Role of the Mitochondrial ABC Transporter MDL1 in the Uptake of Clozapine and Related Molecules into the Yeast *Saccharomyces cerevisiae*

**DOI:** 10.3390/ph17070938

**Published:** 2024-07-13

**Authors:** Chrispian W. Theron, J. Enrique Salcedo-Sora, Justine M. Grixti, Iben Møller-Hansen, Irina Borodina, Douglas B. Kell

**Affiliations:** 1 GeneMill Biofoundry, Liverpool Shared Research Facilities, University of Liverpool, Crown Street, Liverpool L69 7ZB, UK; j.salcedo-sora@liv.ac.uk; 2 Department of Biochemistry, Cell and Systems Biology, Institute of Systems, Molecular and Integrated Biology, University of Liverpool, Crown Street, Liverpool L69 7ZB, UK; 3 The Novo Nordisk Foundation Centre for Biosustainability, Technical University of Denmark, Søltofts Plads 220, 2800 Kongens Lyngby, Denmark

**Keywords:** clozapine, yeast, *S. cerevisiae*, mitochondria, membrane transport, ABC transporter, MDL1, YLR188W

## Abstract

Clozapine is an antipsychotic drug whose accumulation in white cells can sometimes prove toxic; understanding the transporters and alleles responsible is thus highly desirable. We used a strategy in which a yeast (*Saccharomyces cerevisiae*) CRISPR-Cas9 knock-out library was exposed to cytotoxic concentrations of clozapine to determine those transporters whose absence made it more resistant; we also recognised the structural similarity of the fluorescent dye safranin O (also known as safranin T) to clozapine, allowing it to be used as a surrogate marker. Strains lacking the mitochondrial ABC transporter MDL1 (encoded by YLR188W) showed substantial resistance to clozapine. MDL1 overexpression also conferred extra sensitivity to clozapine and admitted a massive increase in the cellular and mitochondrial uptake of safranin O, as determined using flow cytometry and microscopically. Yeast lacking mitochondria showed no such unusual accumulation. Mitochondrial MDL1 is thus the main means of accumulation of clozapine in *S. cerevisiae*. The closest human homologue of *S. cerevisiae* MDL1 is ABCB10.

## 1. Introduction

Clozapine (CLZ) is an effective neuroleptic drug used to treat schizophrenia, particularly when it is refractory to other treatments [1,2,3,4,5]. The chief problem with it is its cellular toxicity [6], and, in particular, there is a clozapine-induced agranulocytosis [7,8,9,10,11,12] that can occur in some 1–4% of those receiving it [13,14,15,16,17]. Consequently, those receiving clozapine must receive regular haematological monitoring [18,19,20,21]. It seems most likely that this is due, at least in part, to a concentrative uptake driven by an allele of the relevant but unknown transporter(s) [22], and indeed, the transporters of most substrates, necessarily involved in drug uptake [23,24,25,26,27], remain unknown [28,29,30]. We note here that there does exist clear and significant evidence for the involvement of such (unknown) transporters in clozapine uptake in mammalian cells [31]. Identifying the relevant transporter(s) and thence their alleles could thereby improve the safety profile of clozapine considerably [31].

One strategy for tackling these kinds of problems is to assess them in experimentally more convenient eukaryotic models such as the yeast *Saccharomyces cerevisiae* (*S. cerevisiae*) [32,33,34], and, in particular, to recognise that if a knock-out library of non-essential genes is available in haploid cells, those genes knocked out that encode transporters of a toxic substrate will enable the organism to become more resistant to it, thereby enabling the identification of the relevant yeast transporter(s) [35]. This strategy has also been applied with great success in mammalian cells [36,37]. To this end, we adopted this and some related strategies to assess those transporters most responsible for the uptake of clozapine into baker’s yeast. The initially unexpected finding here is that a mitochondrial ABC-type transporter, MDL1, is the most clearly identified candidate.

Here, we note the innovative aspects of this paper as follows, given the recognition that in mammalian cells, the important drug, clozapine, possesses at least one transporter [31] but the molecular identities of any of them remain unknown:

- We adapt a comprehensive yeast knock-out library for assessing variation in sensitivity to cytotoxic concentrations of clozapine.
- We exploit modern cheminformatics methods to recognise safranin (and also bilirubin) as a fluorescent analogue of clozapine.
- We determine a mitochondrial location for the chief transporter (MDL1) in terms of physiological effects.
- Using flow cytometry, we determine the massively increased uptake of safranin in cells overexpressing MDL1.
- This mitochondrial transporter is, in fact, and unexpectedly, an ABC transporter acting not as an effluxer but as an influxer (or possibly an antiporter).
- This finding is consistent with other anomalous structural properties of the human homologue (ABCB10) [38] that were seen as being inconsistent with it acting solely as an effluxer.
- The finding of a concentrative uptake via MDL1 provides a simple explanation for the main source of cytotoxicity.
- Overall, the findings underscore the utility of the strategy adopted when seeking transporters for a drug whose transporterome remains unknown.

## 2. Results

### 2.1. Gene Library Screening with Clozapine

**Figure 1:**
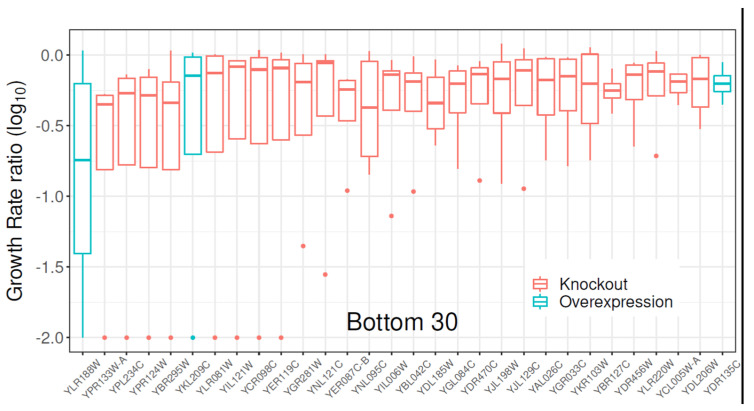
Differential growth of mutated *S. cerevisiae* strains in the presence of 0.1 mM clozapine. The growth rates were normalised to the reference strain, BY4741, represented by a (log) growth rate ratio of 0.0. Strains with transporter proteins deleted are represented in red, while strains overexpressing transporter proteins are represented in aquamarine. The relevant transporter protein affected was used as the strain designation on the *x*-axis. ‘Bottom 30’ refers to the figure representing only the subset of 30 strains with the lowest growth rate ratios relative to that of its reference strain.

The search for membrane transporters potentially involved in clozapine toxicity in the yeast *S. cerevisiae* started with the screening of a collection of strains with modifications to genes encoding proteins expected or known to be membrane transporters (full list in Supplementary Table S1). This collection consisted of 331 gene knock-out strains, with a sub-set (58) of these genes also represented in a smaller collection of overexpressing strains. All strains were exposed to 0.1 mM clozapine, while being cultivated in 96-well plates, with max OD_600_ values of 2.0 in some cases. Lag time, exponential growth rate, and max growth were compiled from four biological replicates (Supplementary Table S1). The distribution of growth rate ratios (the growth rate of each strain over the growth rates in the reference strain, wild-type BY4741) pointed toward the *S. cerevisiae* overexpressing the *YLR188W* gene as being more sensitive to clozapine than the rest of the strains in this collection, including the WT (Figure 1). (Interestingly, the other two overexpressers featuring in Figure 1 also encode transporters: YBR295W/PCA1 is a Cd^++^-transporting P-type ATPase, while YDR135C/YCF1 is a glutathione conjugate transporter.)

The strain overexpressing *YLR188W* (OE) and its corresponding knock-out (KO) variant were selected for further comparison to the wild-type BY4741 (WT) in further studies.

### 2.2. Growth Profile of S. cerevisiae Expressing the Mitochondrial ABC Transporter YLR188W

**Figure 2:**
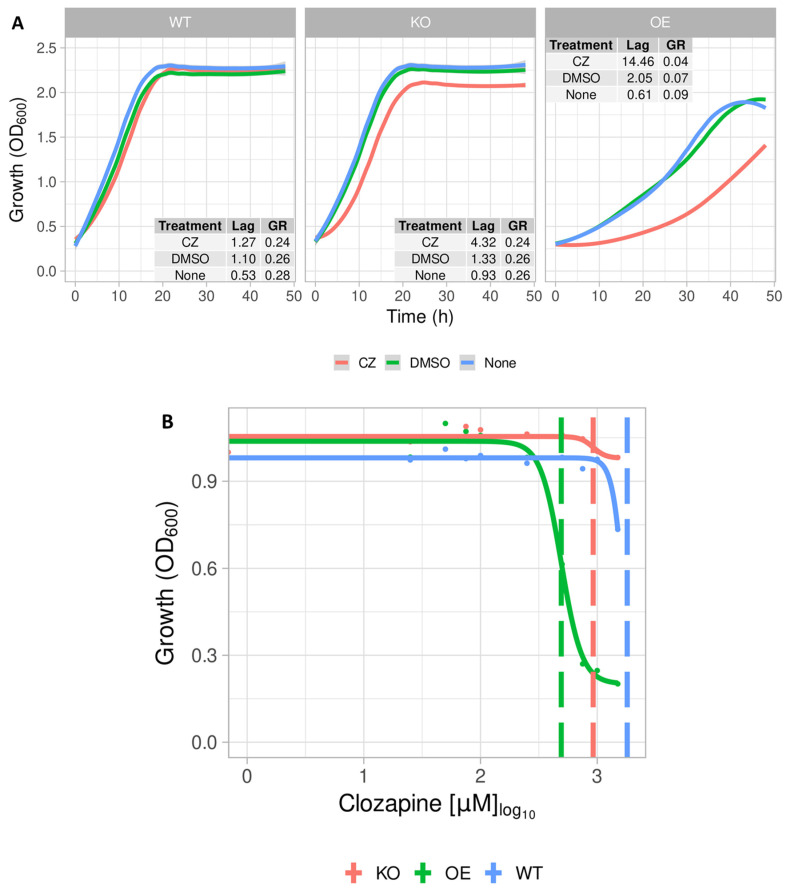
YLR188W (MDL1) sensitivity to clozapine. (**A**) Growth assays illustrating the effect of 0.5 mM CLZ on the reference strain (WT) as well as the YLR188W knock-out (KO) and the strain expressing (OE) YLR188W. (**B**) IC50 determination of the three variants to clozapine.

The overexpression of the mitochondrial membrane protein YLR188W seemed to have a detrimental effect, with yeast cells showing growth rates three-fold lower than those of the WT and the cognate knock-out strain (KO) (Figure 2A,B). Nevertheless, that effect was furthered to a six-fold lower growth rate in the overexpression (OE) strain by the addition of 0.1 mM CLZ (Figure 2A,B). YLR188W (encoding the protein MDL1) encodes for a protein located in the inner membrane of mitochondria in *S. cerevisiae* that is said to be required for the mitochondrial export of peptides with small molecular mass [39,40]. MDL1 also contributes to metal detoxification, probably as part of defensive mechanisms against oxidative stress [41]. The unexpected involvement of this type of protein and its location in this organelle warranted further investigation. Mitochondria and bacteria can accumulate cations to high levels relative to those in the cytoplasm [30,42,43,44,45], potentially offering a straightforward understanding of why a mitochondrial transporter may in fact be seen as the most obvious candidate for cytotoxicity here (there will likely be multiple plasma membrane transporters that do not then show up in this kind of assay [35]).

### 2.3. Detection of Radiolabelled Clozapine in Yeast Cells

**Figure 3:**
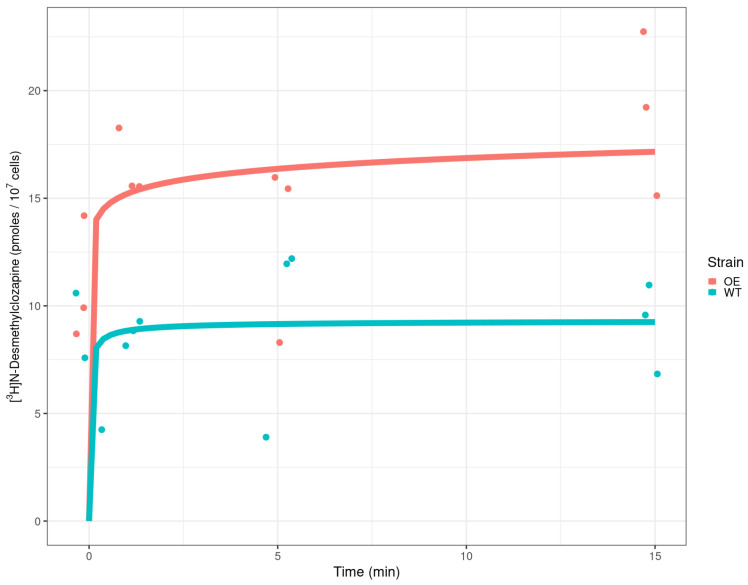
Uptake of [3H]N-Desmethylclozapine in *S. cerevisiae*. WT denotes the reference strain *S. cerevisiae* BY4741, while OE denotes the derived strain carrying the plasmid expressing YLR188W. The uptake of clozapine is reported from triplicates as pmoles per cell 107 cells present in the volume processed for each sample. Both strains seemed to reach the highest levels of uptake from time zero. The cells expressing YLR188W (OE) had consistently higher levels of clozapine in comparison to the reference strain (WT). This difference was similar across the different time points and reached an approximately two-fold difference after 15 min.

An experiment was performed in which the uptake of labelled desmethyl clozapine was assessed in the wild type and an overexpresser of MDL1, with the latter strain clearly taking up more (Figure 3). While this does not exactly mirror the effects on safranin uptake (see lines 166ff), as it is of course a different molecule, it does show an increased uptake by the MDL1 overexpresser. It is also to be assumed that unknown effluxers of clozapines are more effective than are those of safranin.

### 2.4. Cheminformatics That Found Safranin O Similarities with Clozapine

**Figure 4:**
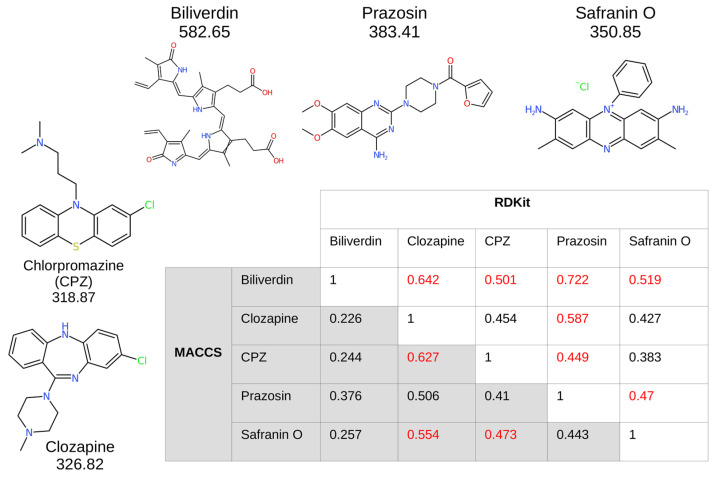
Tanimoto similarity between various of the molecules discussed herein using the MACCS (lower left, grey) and RDKit (upper right, white) encodings. CPZ = chlorpromazine. The exact molecular masses are also given.

Figure 4 shows cheminformatics relationships in the form of Tanimoto similarities between some of the molecules discussed herein, using two classical fingerprint encodings [46] viz. MACCS and RDKit, as provided in the RDKit Fingerprint node of KNIME (http://knime.org/, accessed on 7 July 2024) and widely used by us (e.g., [47,48,49,50]) and others (e.g., [51,52]). Also shown are the relevant structures and the substructural overlaps between chlorpromazine and prazosin [53]. As usual, the values differ both in rank order and magnitude between the two encodings, with red marking which is the greater of the two fingerprint encodings. Interestingly, we note that prazosin, the best competitive inhibitor of clozapine transport [31], is most similar to biliverdin, a known substrate of the ABCB10 transporter [54,55] which is the mammalian homologue of MDL1 [40,56], giving further confidence in our assignment of MDL1 as a clozapine transporter.

### 2.5. Differential Uptake of Safranin O as a Molecular Surrogate for Clozapine

**Figure 5:**
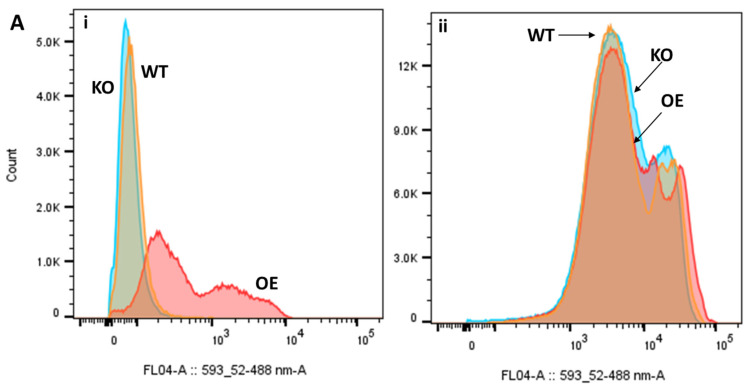

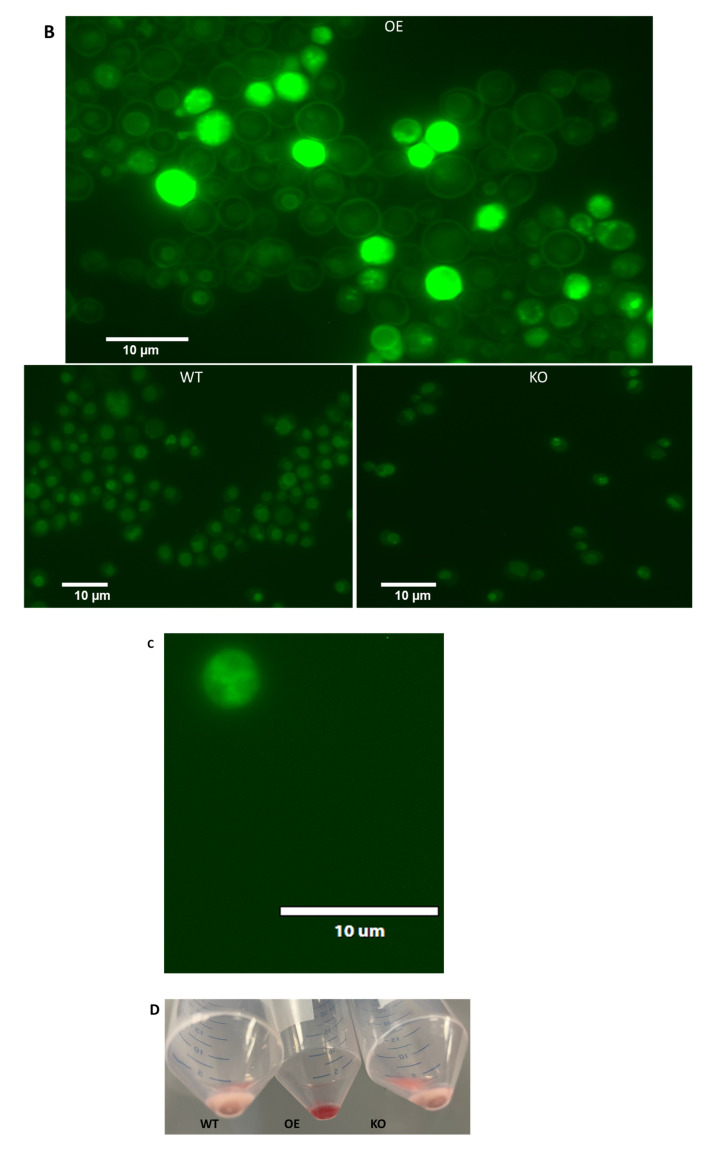
Fluorescence assessment of safranin O uptake by the three test strains. (**A**) Flow cytogram of the reference strain BY4741 (WT, orange), the strain overexpressing YLR188W (OE, red), and YLR188W knock-out strain (KO, blue) after overnight exposure to 100 μM of safranin O in original (**i**) and mitochondria-deficient (Rho^−^, ρ^−^) strains (**ii**). In this specific case, the *y*-axis scales of (**A**) (**i**) and (**ii**) differ as they were performed as parts of separate experiments, in which the voltage settings of the emission filters were notably changed. The differences in fluorescence signals between strains (or lack thereof) are, however, still illustrative. (**B**,**C**) Micrographs of the three strains after staining with 100 μM of safranin O, with (**C**) representing 1000× magnification of the strain overexpressing YLR188W O. (**D**) Image of cell pellets obtained after overnight exposure to 100 μM of safranin O.

As indicated, clozapine and safranin O share a variety of similar structural features (Figure 4). Due to the structural similarities between clozapine and safranin O (Tanimoto score: 0.554), the fluorescence of the latter molecule was used to evaluate the uptake of these molecules. Cultures were treated with safranin O, similarly to how they were exposed to clozapine (Figure 5).

As depicted in Figure 5A(i), the influx of safranin O was considerably (if somewhat heterogeneously) higher in the strains overexpressing YLR188W compared to the other strains investigated, correlating with the growth differences observed in clozapine-treated cultures. In the other two strains, safranin O even appears to be exported during longer-term exposure to the dye (there is precedent [57]) while the YLR188W-overexpressing strain either retains the dye or maintains its import. Mitochondria-deficient (Rho^−^, ρ^−^) variants of the three strains, by contrast, exhibited highly similar fluorescence levels between the three strains, indicative of the absence of the mitochondrial transporter of interest (Figure 5A(ii)).

Although fluorescence levels in the micrographs may not initially appear that different, the difference in background light intensity signifies brighter cellular fluorescence in the overexpressing strain. Interestingly, phenosafranin, a structural derivative of safranin O, was taken up in a similar trend between strains as safranin O (Supplementary Figure S2), complementary to earlier findings [58].

### 2.6. Differential Uptake of Other Molecules

Since ABC transporters are known to be involved in essential cellular processes such as multi-drug resistance, the oxidative stress response, and heme biosynthesis (reviewed by [59,60]), a number of other compounds linked to these processes were tested for uptake by these variant strains.

#### 2.6.1. Chlorpromazine

**Figure 6:**
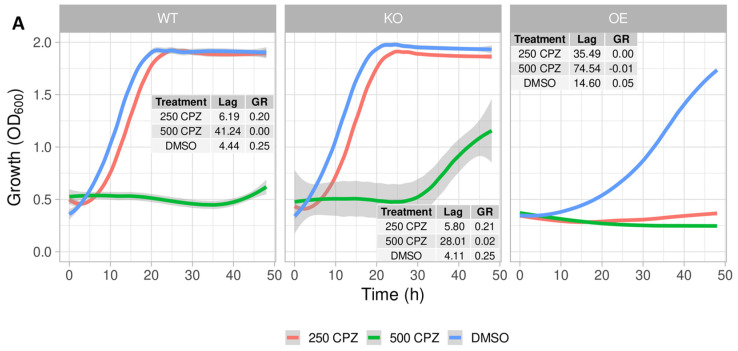

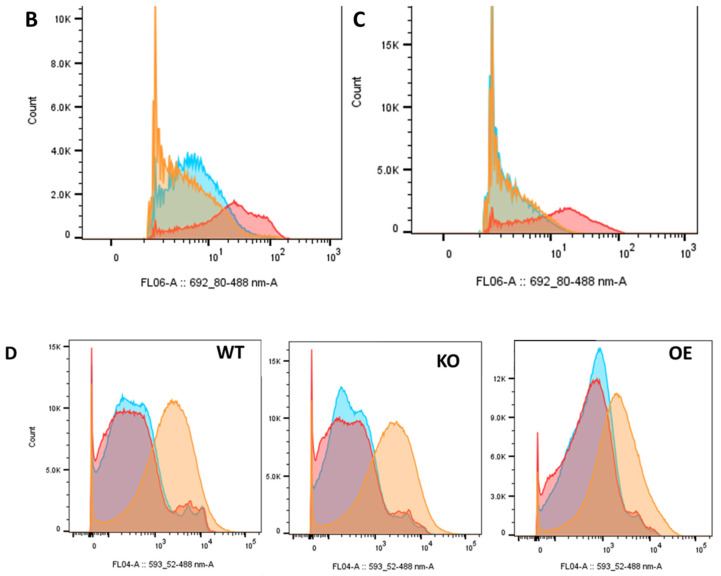

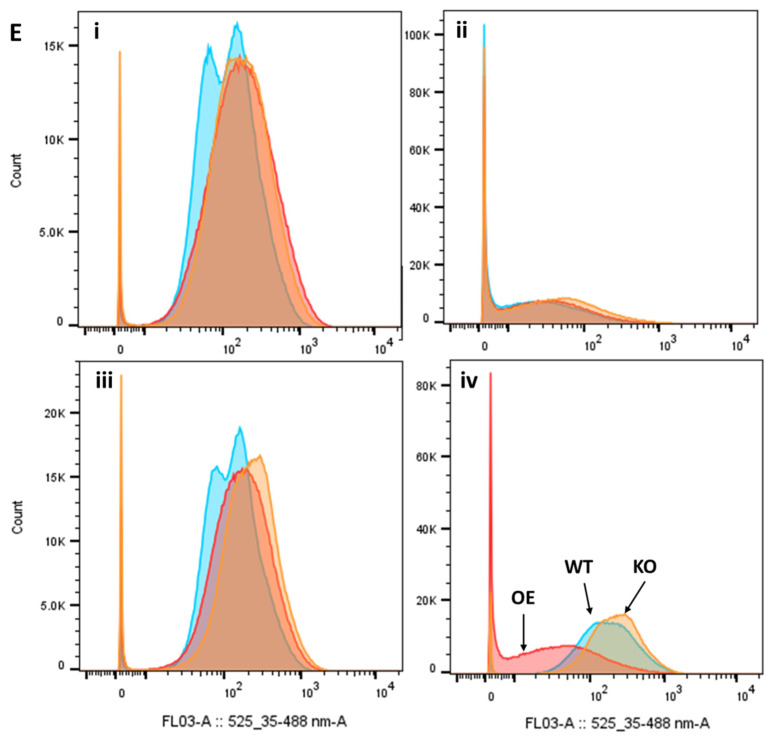
**Treatment of the three strains of this study with other molecules.** (**A**) Growth curves demonstrating the effects of treatment with 250 μM (red line) and 500 μM (green line) of chlorpromazine on the growth of the three test strains, compared to 128 mM DMSO as a control (blue line). (**B**,**C**) Flow cytograms depicting the differential uptake of biliverdin by the WT (blue), OE (red), and KO (orange) strains, without (**B**) and with (**C**) pre-treatment with 500 μM of clozapine overnight. (**D**) Flow cytogram illustrating the effects of 20 min pre-treatment with either 250 μM of chlorpromazine (red), 100 μM of prazosin (orange), or 128 mM DMSO (blue), prior to incubation with safranin O for a further 20 min. (**E**) (**i**)–(**iii**)—Flow cytograms demonstrating the staining of DMSO—(blue), 5 mM—(red) and 10 mM—(orange) H_2_O_2_-treated cultures of the WT (**i**), OE (**ii**), and KO (**iii**) strains with 250 μM of the ROS-responsive dye carboxy-2′,7′-dichlorodihydrofluorescein diacetate (C-H2DCFDA). (**iv**)—Combination of the cytograms of each strain treated with 10 mM H_2_O_2_ for illustrative purposes.

Since ABC transporters are known to be multi-drug transporters, the import of chlorpromazine was also investigated (Figure 6A). With 250 μM of chlorpromazine, the growth of the strain overexpressing YLR188W was notably impeded by the presence of chlorpromazine, while the other two strains were far less affected by its presence. This is, therefore, a strong indicator, as in the case of clozapine, that the negative effect is related to the uptake via this specific transporter.

Chlorpromazine at 250 μM along with clozapine at 500 μM inhibited the growth of all three strains, potentially demonstrating additional off-target effects of the combination (Supplementary Figure S3). The same concentration of chlorpromazine incubated with cultures for 20 min prior to the addition of safranin O appeared to decrease the uptake of safranin O (Figure 6D) only slightly.

#### 2.6.2. Biliverdin

As the centres of cellular respiration, mitochondria are fundamentally linked to heme biosynthesis [61]. Biliverdin, a heme-catabolite, has been shown to be effluxed from mammalian mitochondria by ABCB10 [54,55], the human homologue of MDL1. Biliverdin is also somewhat structurally similar to protoporphyrinogen IX, the last cytoplasmic precursor of heme that is imported into the mitochondrion. The potential of YLR188W to import biliverdin was therefore also analysed. Intriguingly, the YLR188W-overexpressing strain indeed imported significantly more biliverdin than the other two strains investigated did (Figure 6B). Interestingly, bilirubin (a catabolite of biliverdin) was taken up to similar extents by the three strains as biliverdin was (Supplementary Figure S2).

#### 2.6.3. Prazosin

Cultures were incubated for 20 min in the presence of prazosin, followed by incubation for a further 20 min with safranin and analysis of fluorescence. Contrary to the expected inhibition of safranin uptake, safranin uptake instead appeared to be boosted by the prazosin treatment. This effect was, however, the lowest in the overexpressing strain (Figure 6D). Prazosin did not significantly affect growth of any of the strains at concentrations of 100 μM, alone or in the presence of 500 μM of clozapine (Supplementary Figure S3).

#### 2.6.4. C-H2DCFDA

C-H2DCFDA is a redox-responsive fluorogenic probe, which is activated by oxidation by H_2_O_2_ in the presence of an endogenous, intracellular peroxidase [62]. It is hence used as an indicator of reactive oxygen species (ROS) in cells, and therefore by implication an indicator of the state of oxidative stress. As *S. cerevisiae* was shown to be sensitive to 4 mM hydrogen peroxide (H_2_O_2_; [63]), cultures were exposed to either 5 or 10 mM H_2_O_2_ for 30 min to induce oxidative stress. Cells were washed in 0.9% NaCl solution, prior to incubation with 250 μM of C-H2DCFDA for a further 30 min, followed by analysis of the resultant fluorescence. There was an H_2_O_2_-dependent increase in fluorescence in the control and especially the knock-out strains, while the overexpressing strain exhibited significantly lower fluorescence, implying lower ROS levels than in the other strains. These results (Figure 6E) concur with a previous report [41] that MDL1 is indeed involved in protection against oxidative stress by lowering the activities of another mitochondrial transporter, ATM1, which is involved in the transport of iron, which reacts with ROS to make the deadly hydroxyl radical [64,65].

### 2.7. Effect of Clozapine on Safranin O Uptake

**Figure 7:**
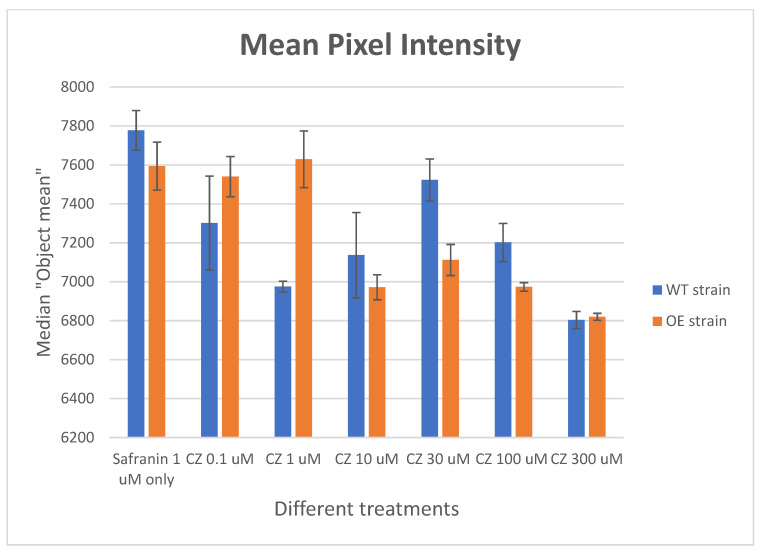
Effect of clozapine concentration on the uptake of safranin O as determined microscopically. Assays were performed as described in Methods as per Figure 5, and in [66], in this case using a 96-well plate. Safranin was present at 1 μM, while clozapine was present at the concentrations indicated. Experiments were performed in triplicate and the error bars indicated SD. *p*-values were as follows: OE 1 vs. 10 μM 0.003, 1 vs. 30 μM 0.004, 1 vs. 100 μM 0.006, 1 vs. 300 μM 0.004.

Finally, although the multiple plasma membrane transporters of clozapine, safranin, and other molecules were not known, we assessed the ability of clozapine to modulate the uptake of safranin, using the microscopic assay detailed in Figure 5B,C. Figure 7 shows that, indeed, clozapine at relatively modest concentrations can inhibit the uptake of safranin O.

## 3. Discussion

The purpose of model organisms “lies in being similar to many organisms without being the same as any other one” [67], and this use of tractable model organisms such as baker’s yeast [33,68] has been at the forefront of post-genomic systems biology. Early studies allowed the discovery of yeast genes encoding transporters for compounds like arginine by mapping the resistance of strains to antimetabolites [69,70]. The availability of libraries of knock-outs and overexpressers, especially using CRISPR-Cas9 [71,72], allows a rapid implementation of the related but wider strategy [35,36] by which transporters may be sought by seeking genome-wide changes in sensitivity and resistance to toxic levels of the substrate of interest. This is what was implemented here.

A chief finding of the present study is that the mitochondrial ABC-type transporter, MDL1, is seemingly responsible for a substantial concentrative uptake of the drug CLZ. Moderately lipophilic cations are widely accumulated by mitochondria [73,74,75,76], though their import transporters in any organism are mainly unknown [42,45] (phosphonium salts can be effluxed efficiently via acrAB-tolC in *E. coli* [77]). We note that MDL2 (YPL270W) is somewhat closely related (https://www.yeastgenome.org/locus/S000004178, accessed 7 July 2024) to MDL1 [78], although actually, it has only 44% sequence identity, and it was represented in the CRISPR-Cas9 library, albeit it is typically not as strongly expressed as MDL1 is. Our proposal is consistent with the facts that MDL1 is imported into mitochondria [79] (MDL2 import is somewhat different [80]) and that MDL1 homologues are known to transport cations [81].

There is other evidence that CLZ targets mitochondria as part of its pharmacological (and possibly off-target) effects [82]. Thus, a chemogenomic study in *S. cerevisiae* showed an interaction between CLZ and the copper-binding protein Cox17 [83], and Cox17 mediates the mitochondrial sequestration of copper in yeast [84,85,86] as well as mammalian cells [87,88]; other proteins transport chaperones that do the same in *E. coli* [89].

Although ABC proteins are normally seen as efflux proteins (e.g., [90,91,92,93], the considerable structural diversity among them (e.g., [38,94]), as well as biochemical evidence, shows that many ABC-type transporters are in fact importers (and/or possibly antiporters) [44,95,96]. The present work provides clear and valuable evidence that MDL1 is another. While there are probably many plasma membrane transporters for CLZ, the present strategy is not sensitive enough to identify them reliably when there are more than a few [35], as the removal of an individual uptake transporter gene typically allows other proteins to “take up the slack” [97]. However, the ability of individual transporters to concentrate substances to toxic levels in intracellular organelles avoids this problem and makes it clear in this case that MDL1 is indeed the chief means of concentrative uptake (and hence the cause of toxicity of CLZ) in yeast, as observed in Figure 1. In this regard, we note that clozapine (as do other lipophilic cations) inhibits complex I and, to a lesser extent, complex III [98].

## 4. Materials and Methods

### 4.1. Chemicals and Medium Components

All medium components were purchased from either Sigma-Aldrich (St. Louis, MO, USA), Merck Millipore (Burlington, MA, USA), Becton Dickinson (BD) (Franklin Lakes, NJ, USA), or Fisher Scientific (Waltham, MA, USA).

Chemical and solvents were purchased from Sigma-Aldrich, except clozapine, which was purchased from Tocris Bioscience (Bio-Techne, Minneapolis, MN, USA). The specific safranin O used in this study is Sigma-Aldrich catalogue number S2255. All chemical (drug and dye) compounds used to treat yeast cells with in this study were dissolved in DMSO for stock solution preparation.

### 4.2. Culture and Treatment of S. cerevisiae Strains

The genomic library of deleted and overexpressed membrane transporters of the *S. cerevisiae* BY4741 strain (MATa his3Δ1 leu2Δ0 met15Δ0 ura3Δ0) that was used in this study was previously created by Wang et al. (2021) [72,99]. A total of 331 knock-out mutants of transporter-encoding genes were cherry picked, and glycerol stocks were stored at −80 °C in 96-well plates.

A small library of 58 transporter gene overexpressions was constructed as follows: The coding sequence of the transporter in question was amplified from genomic DNA of *S. cerevisiae* BY4741, with the primers listed in Supplementary Table S2 (Supplementary Materials). Each PCR fragment containing the transporter of choice was subcloned into a vector at position U2 together with a fragment for the high-expression promoter pTEF1 using USER cloning according to the Easyclone protocol [100]. The vector for integration was amplified using the primer pair listed in Supplementary Table S2 (Supplementary Materials). The transporters were individually integrated at the genomic site XII-2 using CRISPR-Cas9 integration with KanMX as the dominant marker, as described previously [101]. The integration at the correct loci was confirmed by PCR according to the integration site.

Pre-cultures of yeast strains were grown in yeast rich medium (YPG) (yeast extract (10 g·L^−1^), Bacto peptone (20 g·L^−1^), and galactose (20 g·L^−1^)). Pre-cultures were used to inoculate minimal medium [Difco^TM^ yeast nitrogen base (6.7 g·L^−1^), yeast synthetic drop-out medium without histidine, leucine, tryptophan, and uracil (1.4 g·L^−1^); galactose (20 g·L^−1^), supplemented with histidine, uracil, tryptophan (all (76 mg·L^−1^)); and leucine (0.38 g·L^−1^)] for working cultures. Stock solutions of clozapine (CLZ) were prepared using dimethyl sulfoxide (DMSO).

Growth inhibitory assays to calculate the IC_50_ of CLZ in the *S. cerevisiae* reference strain BY4741 were carried out in microtitration assays. A total of 50 µL of two-fold dilutions of CLZ was transferred to 96-well plates to which 50 µL of fresh yeast culture was added. Endpoint reads of the media turbidity at 600 nm (OD_600_) were taken after 24 h at 30 °C.

The CLZ exposure growth assays for the genetic library screening were carried out by seeding glycerol stocks into 96-well plates with 50 µL of yeast complex media with galactose (YPG) containing 200 µg/mL of geneticin. The sealed plates were then incubated overnight at 30 °C. The overnight cultures were diluted 1000-fold in 50 µL of fresh YPG without geneticin in polystyrene 96-well plates with transparent bottoms (Sigma M6936-40EA). CLZ was added to a final concentration of 100 μM. Control wells only had 0.5% v/v DMSO added. *S. cerevisiae* reference strain BY4741 was included as the “wild-type” control (WT). These plates were heat-sealed with plastic covers and incubated for 24 h at 30 °C with 200 rpm shaking, with absorbance measurements at 25 min intervals. Experimental work with multiple multi-well plates was carried out in a customised robotic station built by Analytik-Jena AG (Thuringia, Germany).

The inhibitory concentrations of clozapine that reduced yeast growth by half (IC_50_) were calculated with the four-parameter logistic model, as implemented in the R package *drc* (URL https://www.R-project.org/, accessed on 7 July 2024). The growth rates in the exponential phase of batch cultures were extracted from the curve fitting of cell growth with a parametric non-linear least-squares method. The lag times were calculated using a linear fitting of the log-transformed data [102,103]. Both approaches were carried out as implemented in the R package *growthrates*.

The strains used for further studies were the wild-type BY4741 as a reference/control strain (WT), a strain overexpressing YLR188W (OE), and a strain with YLR188W deleted (KO). These studies included further growth studies on clozapine and chlorpromazine (250 μM); and monitoring fluorescence after treatment with safranin (100 μM), prazosin (100 μM), biliverdin (10 μM), or carboxy-2′,7′-dichlorodihydrofluorescein diacetate (C-HDCFDA, 250 μM) (Section 2.5).

Mitochondria-deficient variants of the selected strains were prepared by treating the strains with 10 µg/mL ethidium bromide overnight in the dark. Mitochondrial loss was confirmed by the loss of ability to grow on the non-fermentable carbon source, glycerol (Supplementary Figure S1; [104]).

### 4.3. Detection of Radiolabelled Clozapine in Yeast Cells

Wild-type *S. cerevisiae* and *S. cerevisiae* overexpressing the MDL1 transporter were exposed to [3H] N-Desmethylclozapine (Moravek Inc., Brea, CA, USA, Lot 593-017-0021-A-20220815-JPL) at a final activity of 1 μCi. Cells were resuspended to an OD600 1 in transport medium (TM) consisting of YNB, galactose, unlabelled clozapine (Bio-Techne, product 0444/50) at a final concentration of 400 μM, and radiolabelled clozapine. Aliquots of 1 mL were taken at time points 0, 1, 5, and 15 min and added to ice-cold TM (without labelled and unlabelled CLZ) to stop the reaction. Cells were filtered using Whatman 3 filter paper (diameter: 23 mm, cat 1003-323) on a sampling manifold. Cells were washed with an additional 4 mL of TM, followed by a second round of filtration, after which, filter papers were transferred to scintillation tubes, followed by the addition of 4 mL of OptiPhase HiSafe 3 (PerkinElmer, Waltham, MA, USA) and vortexing, before samples were run on a Tri-Carb^®^ 2910TR liquid scintillation analyser (Perkin Elmer). The final concentration of clozapine per sample (pmoles per 10^7^ cells, Figure 3) was calculated from a specific activity of 2.1 Ci/mmol and an estimated 50% efficiency for the Tri-Carb^®^ 2910TR liquid scintillation analyser.

### 4.4. Cheminformatics That Assessed Safranin O Similarities with Clozapine

As part of an extensive study of drug-substrate similarities (e.g., [25,47,50,105,106]), we earlier established a number of novel deep learning strategies including a variational autoencoder [53] and a transformer [107] that indicated the closeness of the structures of clozapine and safranin O. As with other organic cations, safranin has long been known to be accumulated in an energy-dependent manner by respiring mitochondria [108] and was thus of interest, as it might be a surrogate for the accumulation of clozapine.

### 4.5. Fluorescence Analysis

Overnight cultures were standardised to an OD_600_ of 1 by resuspension in 0.9% sodium chloride solution. Safranin O was then added to a final concentration of 100 μM (from DMSO stock), and samples were incubated at 30 °C for 20 min or alternatively overnight.

Flow cytometry was performed using a Bio-Rad ZE5 cell analyser. The flow rate was set to 1 µL/s and samples were standardised by collecting the same number of events per sample. Safranin O fluorescence was excited using the 488 nm laser and measured using the 593/52 nm filter. Biliverdin fluorescence was excited using the 488 nm laser and measured using the 692/80 nm filter. C-H_2_DCFDA fluorescence was excited using the 488 nm laser and measured using the 525/35 nm filter.

Fluorescence microscopy was performed using an EVOS FL auto microscope (Life technologies, Carlsbad, CA, USA) using the GFP filter, on samples prepared in the same manner as for flow cytometry.

## 5. Conclusions

The present findings add weight to the strategies of (i) seeking transporters by assessing genetically encoded variations in the toxicity of candidate substrates and (ii) using flow cytometry as a surrogate for high-throughput screening [43,44,109]. Now, we are well poised to exploit the same method for other substrates and in other cellular systems, for both drug uptake and in cellular factories [30,110,111].

## Data Availability

Relevant data are given in the Supplementary Materials described above.

## Supplementary Materials

The following supporting information can be downloaded at https://www.mdpi.com/article/10.3390/ph17070938/s1: Table S1: Table_Yeast_DTU_Growth_assays_C. Table S2: Primers used for the construction of the transporter libraries. Figure S1: Growth profiles of triplicate cultures of the reference strain BY4741 (Ref), the overexpressing strain (OE) and the knockout strain (KO) under different conditions. Growth of original strains on glycerol as carbon source (**A**); were compared to mitochondrially deficient (Rho^−^, ρ^−^) versions of each strain, using glucose (**B**) and glycerol (**C**) as carbon sources. Figure S2: Differential uptake of (**A**) safranin O vs Phenosafranin; and (**B**) bilirubin, by the reference strain BY4741 (orange), the strain overexpressing YLR188W (red) and the YLR188W-knockout strain (blue). Figure S3: Effects of different drugs and drug combinations on the growth of the reference strain BY4741 (WT), the strain overexpressing YLR188W (OE) and the YLR188W-knockout strain (KO). CPZ – chlorpromazine, CZ – clozapine, DMSO – dimethyl sulfoxide, PZ – prazosin.

## References

[1] Correll C.U., Agid O., Crespo-Facorro B., de Bartolomeis A., Fagiolini A., Seppälä N., Howes O.D. (2022). A Guideline and Checklist for Initiating and Managing Clozapine Treatment in Patients with Treatment-Resistant Schizophrenia. CNS Drugs.

[2] Hatano M., Kamei H., Takeuchi I., Gomi K., Sakakibara T., Hotta S., Esumi S., Tsubouchi K., Shimizu Y., Yamada S. (2023). Long-term outcomes of delayed clozapine initiation in treatment-resistant schizophrenia: A multicenter retrospective cohort study. BMC Psychiatry.

[3] Siskind D., McCartney L., Goldschlager R., Kisely S. (2016). Clozapine v. first- and second-generation antipsychotics in treatment-refractory schizophrenia: Systematic review and meta-analysis. Br. J. Psychiatry.

[4] Qubad M., Bittner R.A. (2023). Second to none: Rationale, timing, and clinical management of clozapine use in schizophrenia. Ther. Adv. Psychopharmacol..

[5] Freibüchler A., Seifert R. (2024). Analysis of clinical studies on clozapine from 2012–2022. Naunyn-Schmiedeberg’s Arch. Pharmacol..

[6] Tanzer T., Pham B., Warren N., Barras M., Kisely S., Siskind D. (2024). Overcoming clozapine’s adverse events: A narrative review of systematic reviews and meta-analyses. Expert. Opin. Drug Saf..

[7] Crilly J. (2007). The history of clozapine and its emergence in the US market: A review and analysis. Hist. Psychiatry.

[8] Idänpään-Heikkilä J., Alhava E., Olkinuora M., Palva I.P. (1977). Agranulocytosis during treatment with chlozapine. Eur. J. Clin. Pharmacol..

[9] De Berardis D., Rapini G., Olivieri L., Di Nicola D., Tomasetti C., Valchera A., Fornaro M., Di Fabio F., Perna G., Di Nicola M. (2018). Safety of antipsychotics for the treatment of schizophrenia: A focus on the adverse effects of clozapine. Ther. Adv. Drug Saf..

[10] Skokou M., Karavia E.A., Drakou Z., Konstantinopoulou V., Kavakioti C.A., Gourzis P., Kypreos K.E., Andreopoulou O. (2022). Adverse Drug Reactions in Relation to Clozapine Plasma Levels: A Systematic Review. Pharmaceuticals.

[11] Mijovic A., MacCabe J.H. (2020). Clozapine-induced agranulocytosis. Ann. Hematol..

[12] Alalawi A., Albalawi E., Aljohani A., Almutairi A., Alrehili A., Albalawi A., Aldhafiri A. (2024). Decoding Clozapine-Induced Agranulocytosis: Unraveling Interactions and Mitigation Strategies. Pharmacy.

[13] Haddad P.M., Sharma S.G. (2007). Adverse effects of atypical antipsychotics: Differential risk and clinical implications. CNS Drugs.

[14] Alvir J.M., Lieberman J.A., Safferman A.Z., Schwimmer J.L., Schaaf J.A. (1993). Clozapine-induced agranulocytosis. Incidence and risk factors in the United States. N. Engl. J. Med..

[15] Leucht S., Corves C., Arbter D., Engel R.R., Li C., Davis J.M. (2009). Second-generation versus first-generation antipsychotic drugs for schizophrenia: A meta-analysis. Lancet.

[16] Li K.J., Gurrera R.J., Delisi L.E. (2018). Potentially fatal outcomes associated with clozapine. Schizophr. Res..

[17] Wiciński M., Węclewicz M.M. (2018). Clozapine-induced agranulocytosis/granulocytopenia: Mechanisms and monitoring. Curr. Opin. Hematol..

[18] Fenton C., Kang C. (2023). Clozapine is the approved option in treatment-resistant schizophrenia and requires careful management. Drugs Ther. Perspect..

[19] Nielsen J., Young C., Ifteni P., Kishimoto T., Xiang Y.T., Schulte P.F.J., Correll C.U., Taylor D. (2016). Worldwide Differences in Regulations of Clozapine Use. CNS Drugs.

[20] Kar N., Barreto S., Chandavarkar R. (2016). Clozapine Monitoring in Clinical Practice: Beyond the Mandatory Requirement. Clin. Psychopharmacol. Neurosci..

[21] Kar A., Nutting T., Ikram M., Sullivan C. (2023). The clozapine conundrum: Navigating neutropenia and the pursuit of effective care in treatment-resistant schizophrenia. Int. J. Psychiatry Med..

[22] Bergemann N., Abu-Tair F., Aderjan R., Kopitz J. (2007). High clozapine concentrations in leukocytes in a patient who developed leukocytopenia. Prog. Neuropsychopharmacol. Biol. Psychiatry.

[23] Kell D.B., Oliver S.G. (2014). How drugs get into cells: Tested and testable predictions to help discriminate between transporter-mediated uptake and lipoidal bilayer diffusion. Front. Pharmacol..

[24] Roberts A.G. (2021). The Structure and Mechanism of Drug Transporters. Methods Mol. Biol..

[25] Kell D.B., Samanta S., Swainston N. (2020). Deep learning and generative methods in cheminformatics and chemical biology: Navigating small molecule space intelligently. Biochem. J..

[26] Markowicz-Piasecka M., Darlak P., Markiewicz A., Sikora J., Kumar Adla S., Bagina S., Huttunen K.M. (2022). Current approaches to facilitate improved drug delivery to the central nervous system. Eur. J. Pharm. Biopharm..

[27] Alam S., Doherty E., Ortega-Prieto P., Arizanova J., Fets L. (2023). Membrane transporters in cell physiology, cancer metabolism and drug response. Dis. Model. Mech..

[28] César-Razquin A., Snijder B., Frappier-Brinton T., Isserlin R., Gyimesi G., Bai X., Reithmeier R.A., Hepworth D., Hediger M.A., Edwards A.M. (2015). A call for systematic research on solute carriers. Cell.

[29] Superti-Furga G., Lackner D., Wiedmer T., Ingles-Prieto A., Barbosa B., Girardi E., Goldman U., Gürtl B., Klavins K., Klimek C. (2020). The RESOLUTE consortium: Unlocking SLC transporters for drug discovery. Nat. Rev. Drug Discov..

[30] Kell D.B. (2021). The transporter-mediated cellular uptake and efflux of pharmaceutical drugs and biotechnology products: How and why phospholipid bilayer transport is negligible in real biomembranes. Molecules.

[31] Dickens D., Rädisch S., Chiduza G.N., Giannoudis A., Cross M.J., Malik H., Schaeffeler E., Sison-Young R.L., Wilkinson E.L., Goldring C.E. (2018). Cellular uptake of the atypical antipsychotic clozapine is a carrier-mediated process. Mol. Pharm..

[32] Oliver S.G. (1997). Yeast as a navigational aid in genome analysis. Microbiology.

[33] Oliver S.G., Winson M.K., Kell D.B., Baganz F. (1998). Systematic functional analysis of the yeast genome. Trends Biotechnol..

[34] Herrgård M.J., Swainston N., Dobson P., Dunn W.B., Arga K.Y., Arvas M., Blüthgen N., Borger S., Costenoble R., Heinemann M. (2008). A consensus yeast metabolic network obtained from a community approach to systems biology. Nat. Biotechnol..

[35] Lanthaler K., Bilsland E., Dobson P., Moss H.J., Pir P., Kell D.B., Oliver S.G. (2011). Genome-wide assessment of the carriers involved in the cellular uptake of drugs: A model system in yeast. BMC Biol..

[36] Winter G.E., Radic B., Mayor-Ruiz C., Blomen V.A., Trefzer C., Kandasamy R.K., Huber K.V.M., Gridling M., Chen D., Klampfl T. (2014). The solute carrier SLC35F2 enables YM155-mediated DNA damage toxicity. Nat. Chem. Biol..

[37] Girardi E., César-Razquin A., Lindinger S., Papakostas K., Lindinger S., Konecka J., Hemmerich J., Kickinger S., Kartnig F., Gürtl B. (2020). A widespread role for SLC transmembrane transporters in resistance to cytotoxic drugs. Nat. Chem. Biol..

[38] Shintre C.A., Pike A.C., Li Q., Kim J.I., Barr A.J., Goubin S., Shrestha L., Yang J., Berridge G., Ross J. (2013). Structures of ABCB10, a human ATP-binding cassette transporter in apo- and nucleotide-bound states. Proc. Natl. Acad. Sci. USA.

[39] Young L., Leonhard K., Tatsuta T., Trowsdale J., Langer T. (2001). Role of the ABC transporter Mdl1 in peptide export from mitochondria. Science.

[40] Herget M., Tampé R. (2007). Intracellular peptide transporters in human—Compartmentalization of the “peptidome”. Pflugers Arch..

[41] Chloupková M., LeBard L.S., Koeller D.M. (2003). MDL1 is a high copy suppressor of ATM1: Evidence for a role in resistance to oxidative stress. J. Mol. Biol..

[42] Radi M.S., Munro L.J., Sora J.E.S., Kim S.H., Feist A.M., Kell D.B. (2022). Understanding functional redundancy and promiscuity of multidrug transporters in *E. coli* under lipophilic cation stress. Membranes.

[43] Salcedo-Sora J.E., Jindal S., O’Hagan S., Kell D.B. (2021). A palette of fluorophores that are differentially accumulated by wild-type and mutant strains of *Escherichia coli*: Surrogate ligands for bacterial membrane transporters. Microbiology.

[44] Jindal S., Yang L., Day P.J., Kell D.B. (2019). Involvement of multiple influx and efflux transporters in the accumulation of cationic fluorescent dyes by *Escherichia coli*. BMC Microbiol..

[45] Kell D.B. (2021). A protet-based, protonic charge transfer model of energy coupling in oxidative and photosynthetic phosphorylation. Adv. Micr Physiol..

[46] Gasteiger J. (2003). Handbook of Chemoinformatics: From Data to Knowledge.

[47] O’Hagan S., Swainston N., Handl J., Kell D.B. (2015). A ‘rule of 0.5’ for the metabolite-likeness of approved pharmaceutical drugs. Metabolomics.

[48] O’Hagan S., Kell D.B. (2015). The KNIME workflow environment and its applications in Genetic Programming and machine learning. Genetic Progr Evol. Mach..

[49] O’Hagan S., Kell D.B. (2017). Analysis of drug-endogenous human metabolite similarities in terms of their maximum common substructures. J. Cheminform.

[50] O’Hagan S., Kell D.B. (2017). Consensus rank orderings of molecular fingerprints illustrate the ‘most genuine’ similarities between marketed drugs and small endogenous human metabolites, but highlight exogenous natural products as the most important ‘natural’ drug transporter substrates. Admet Dmpk.

[51] Mazanetz M.P., Marmon R.J., Reisser C.B.T., Morao I. (2012). Drug discovery applications for KNIME: An open source data mining platform. Curr. Top. Med. Chem..

[52] Roughley S.D. (2020). Five Years of the KNIME Vernalis Cheminformatics Community Contribution. Curr. Med. Chem..

[53] Samanta S., O’Hagan S., Swainston N., Roberts T.J., Kell D.B. (2020). VAE-Sim: A novel molecular similarity measure based on a variational autoencoder. Molecules.

[54] Cao S., Yang Y., He L., Hang Y., Yan X., Shi H., Wu J., Ouyang Z. (2023). Cryo-EM structures of mitochondrial ABC transporter ABCB10 in apo and biliverdin-bound form. Nat. Commun..

[55] Shum M., Shintre C.A., Althoff T., Gutierrez V., Segawa M., Saxberg A.D., Martinez M., Adamson R., Young M.R., Faust B. (2021). ABCB10 exports mitochondrial biliverdin, driving metabolic maladaptation in obesity. Sci. Transl. Med..

[56] Schaedler T.A., Faust B., Shintre C.A., Carpenter E.P., Srinivasan V., van Veen H.W., Balk J. (2015). Structures and functions of mitochondrial ABC transporters. Biochem. Soc. Trans..

[57] Grixti J., O’Hagan S., Day P.J., Kell D.B. (2017). Enhancing drug efficacy and therapeutic index through cheminformatics-based selection of small molecule binary weapons that improve transporter-mediated targeting: A cytotoxicity system based on gemcitabine. Front. Pharmacol..

[58] Brenner S. (1953). Supravital staining of mitochondria with phenosafranin dyes. Biochim. Biophys. Acta.

[59] Jungwirth H., Kuchler K. (2006). Yeast ABC transporters—A tale of sex, stress, drugs and aging. FEBS Lett..

[60] Zutz A., Gompf S., Schagger H., Tampé R. (2009). Mitochondrial ABC proteins in health and disease. Biochim. Biophys. Acta.

[61] Ryter S.W. (2021). Significance of Heme and Heme Degradation in the Pathogenesis of Acute Lung and Inflammatory Disorders. Int. J. Mol. Sci..

[62] Sadler J.C., Currin A., Kell D.B. (2018). Ultra-high-throughput functional enrichment of large monoamine oxidase (MAO-N) libraries by fluorescence activated cell sorting. Analyst.

[63] Tran K., Green E.M. (2019). Assessing Yeast Cell Survival Following Hydrogen Peroxide Exposure. Bio Protoc..

[64] Kell D.B. (2009). Iron behaving badly: Inappropriate iron chelation as a major contributor to the aetiology of vascular and other progressive inflammatory and degenerative diseases. BMC Med. Genom..

[65] Kell D.B. (2010). Towards a unifying, systems biology understanding of large-scale cellular death and destruction caused by poorly liganded iron: Parkinson’s, Huntington’s, Alzheimer’s, prions, bactericides, chemical toxicology and others as examples. Arch. Toxicol..

[66] Grixti J.M., Theron C.W., Salcedo-Sora J.E., Pretorius E., Kell D.B. (2024). Automated microscopic measurement of fibrinaloid microclots and their degradation by nattokinase, the main natto protease. bioRxiv.

[67] Kell D.B., Knowles J.D., Szallasi Z., Stelling J., Periwal V. (2006). The role of modeling in systems biology. System Modeling in Cellular Biology: From Concepts to Nuts and Bolts.

[68] Oliver S.G. (2002). Functional genomics: Lessons from yeast. Philos. Trans. R. Soc. Lond. B Biol. Sci..

[69] Grenson M., Mousset M., Wiame J.M., Bechet J. (1966). Multiplicity of the amino acid permeases in *Saccharomyces cerevisiae*. I. Evidence for a specific arginine-transporting system. Biochim. Biophys. Acta.

[70] Hoffmann W. (1985). Molecular characterization of the CAN1 locus in *Saccharomyces cerevisiae*. A transmembrane protein without N-terminal hydrophobic signal sequence. J. Biol. Chem..

[71] Stovicek V., Holkenbrink C., Borodina I. (2017). CRISPR/Cas system for yeast genome engineering: Advances and applications. FEMS Yeast Res..

[72] Wang G., Møller-Hansen I., Babaei M., D’Ambrosio V., Christensen H.B., Darbani B., Jensen M.K., Borodina I. (2021). Transportome-wide engineering of *Saccharomyces cerevisiae*. Metab. Eng..

[73] Tedeschi H. (1981). The transport of cations in mitochondria. Biochim. Biophys. Acta.

[74] Murphy M.P., Smith R.A.J. (2007). Targeting antioxidants to mitochondria by conjugation to lipophilic cations. Annu. Rev. Pharmacol. Toxicol..

[75] Murphy M.P. (2008). Targeting lipophilic cations to mitochondria. Biochim. Biophys. Acta.

[76] Ross M.F., Da Ros T., Blaikie F.H., Prime T.A., Porteous C.M., Severina I.I., Skulachev V.P., Kjaergaard H.G., Smith R.A.J., Murphy M.P. (2006). Accumulation of lipophilic dications by mitochondria and cells. Biochem. J..

[77] Li X.Z., Plésiat P., Nikaido H. (2015). The challenge of efflux-mediated antibiotic resistance in Gram-negative bacteria. Clin. Microbiol. Rev..

[78] Dean M., Allikmets R., Gerrard B., Stewart C., Kistler A., Shafer B., Michaelis S., Strathern J. (1994). Mapping and sequencing of two yeast genes belonging to the ATP-binding cassette superfamily. Yeast.

[79] Melin J., Kilisch M., Neumann P., Lytovchenko O., Gomkale R., Schendzielorz A., Schmidt B., Liepold T., Ficner R., Jahn O. (2015). A presequence-binding groove in Tom70 supports import of Mdl1 into mitochondria. Biochim. Biophys. Acta.

[80] Park K., Jung S.J., Kim H., Kim H. (2014). Mode of membrane insertion of individual transmembrane segments in Mdl1 and Mdl2, multi-spanning mitochondrial ABC transporters. FEBS Lett..

[81] Miljkovic M., Seguin A., Jia X., Cox J.E., Catrow J.L., Bergonia H., Phillips J.D., Stephens W.Z., Ward D.M. (2023). Loss of the mitochondrial protein Abcb10 results in altered arginine metabolism in MEL and K562 cells and nutrient stress signaling through ATF4. J. Biol. Chem..

[82] Casademont J., Garrabou G., Miro O., Lopez S., Pons A., Bernardo M., Cardellach F. (2007). Neuroleptic treatment effect on mitochondrial electron transport chain: Peripheral blood mononuclear cells analysis in psychotic patients. J. Clin. Psychopharmacol..

[83] Hillenmeyer M.E., Ericson E., Davis R.W., Nislow C., Koller D., Giaever G. (2010). Systematic analysis of genome-wide fitness data in yeast reveals novel gene function and drug action. Genome Biol..

[84] Horng Y.C., Cobine P.A., Maxfield A.B., Carr H.S., Winge D.R. (2004). Specific copper transfer from the Cox17 metallochaperone to both Sco1 and Cox11 in the assembly of yeast cytochrome C oxidase. J. Biol. Chem..

[85] Abajian C., Yatsunyk L.A., Ramirez B.E., Rosenzweig A.C. (2004). Yeast cox17 solution structure and Copper(I) binding. J. Biol. Chem..

[86] Shi H., Jiang Y., Yang Y., Peng Y., Li C. (2021). Copper metabolism in *Saccharomyces cerevisiae*: An update. Biometals.

[87] Voronova A., Meyer-Klaucke W., Meyer T., Rompel A., Krebs B., Kazantseva J., Sillard R., Palumaa P. (2007). Oxidative switches in functioning of mammalian copper chaperone Cox17. Biochem. J..

[88] Chen J., Jiang Y., Shi H., Peng Y., Fan X., Li C. (2020). The molecular mechanisms of copper metabolism and its roles in human diseases. Pflugers Arch..

[89] Salcedo-Sora J.E., Robison A.T.R., Zaengle-Barone J., Franz K.J., Kell D.B. (2021). Membrane transporters involved in the antimicrobial activities of pyrithione in *Escherichia coli*. Molecules.

[90] Baylay A.J., Ivens A., Piddock L.J. (2015). A novel gene amplification causes upregulation of the PatAB ABC transporter and fluoroquinolone resistance in *Streptococcus pneumoniae*. Antimicrob. Agents Chemother..

[91] Alam A., Locher K.P. (2023). Structure and Mechanism of Human ABC Transporters. Annu. Rev. Biophys..

[92] Chen Z., Shi T., Zhang L., Zhu P., Deng M., Huang C., Hu T., Jiang L., Li J. (2016). Mammalian drug efflux transporters of the ATP binding cassette (ABC) family in multidrug resistance: A review of the past decade. Cancer Lett..

[93] Orelle C., Mathieu K., Jault J.M. (2019). Multidrug ABC transporters in bacteria. Res. Microbiol..

[94] Thomas C., Aller S.G., Beis K., Carpenter E.P., Chang G., Chen L., Dassa E., Dean M., Duong Van Hoa F., Ekiert D. (2020). Structural and functional diversity calls for a new classification of ABC transporters. FEBS Lett..

[95] Ter Beek J., Guskov A., Slotboom D.J. (2014). Structural diversity of ABC transporters. J. Gen. Physiol..

[96] Lewinson O., Livnat-Levanon N. (2017). Mechanism of Action of ABC Importers: Conservation, Divergence, and Physiological Adaptations. J. Mol. Biol..

[97] Mendes P., Girardi E., Superti-Furga G., Kell D.B. (2001). Why most transporter mutations that cause antibiotic resistance are to efflux pumps rather than to import transporters. bioRxiv.

[98] Elmorsy E., Smith P.A. (2015). Bioenergetic disruption of human micro-vascular endothelial cells by antipsychotics. Biochem. Biophys. Res. Commun..

[99] Winzeler E.A., Shoemaker D.D., Astromoff A., Liang H., Anderson K., Andre B., Bangham R., Benito R., Boeke J.D., Bussey H. (1999). Functional characterization of the *S. cerevisiae* genome by gene deletion and parallel analysis. Science.

[100] Stovicek V., Borja G.M., Forster J., Borodina I. (2015). EasyClone 2.0: Expanded toolkit of integrative vectors for stable gene expression in industrial Saccharomyces cerevisiae strains. J. Ind. Microbiol. Biotechnol..

[101] Jessop-Fabre M.M., Jakociunas T., Stovicek V., Dai Z.J., Jensen M.K., Keasling J.D., Borodina I. (2016). EasyClone-MarkerFree: A vector toolkit for marker-less integration of genes into *Saccharomyces cerevisiae* via CRISPR-Cas9. Biotechnol. J..

[102] Hall B.G., Acar H., Nandipati A., Barlow M. (2014). Growth rates made easy. Mol. Biol. Evol..

[103] Vaas L.A., Sikorski J., Michael V., Göker M., Klenk H.P. (2012). Visualization and curve-parameter estimation strategies for efficient exploration of phenotype microarray kinetics. PLoS ONE.

[104] Misonou Y., Kikuchi M., Sato H., Inai T., Kuroiwa T., Tanaka K., Miyakawa I. (2014). Aldehyde dehydrogenase, Ald4p, is a major component of mitochondrial fluorescent inclusion bodies in the yeast *Saccharomyces cerevisiae*. Biol. Open.

[105] O’Hagan S., Kell D.B. (2016). MetMaxStruct: A Tversky-similarity-based strategy for analysing the (sub)structural similarities of drugs and endogenous metabolites. Front. Pharmacol..

[106] O’Hagan S., Kell D.B. (2020). Structural similarities between some common fluorophores used in biology, marketed drugs, endogenous metabolites, and natural products. Marine Drugs.

[107] Shrivastava A.D., Kell D.B. (2021). FragNet, a contrastive learning-based transformer model for clustering, interpreting, visualising and navigating chemical space. Molecules.

[108] Åkerman K.E.O., Wikström M.K.F. (1976). Safranine as a probe of the mitochondrial membrane potential. FEBS Lett..

[109] Davey H.M., Kell D.B. (1996). Flow cytometry and cell sorting of heterogeneous microbial populations: The importance of single-cell analysis. Microbiol. Rev..

[110] van der Hoek S.A., Borodina I. (2020). Transporter engineering in microbial cell factories: The ins, the outs, and the in-betweens. Curr. Opin. Biotechnol..

[111] Kell D.B., El-Mansi E.M.T., Nielsen J., Mousdale D., Allman T., Carlson R. (2019). Control of metabolite efflux in microbial cell factories: Current advances and future prospects. Fermentation Microbiology and Biotechnology.

