# Supplementary material for: Evidence for the Role of the Mitochondrial ABC Transporter MDL1 in the Uptake of Clozapine and Related Molecules into the Yeast Saccharomyces cerevisiae
Source: Pharmaceuticals (Basel). 2024 Jul 13;17(7):938. doi: 10.3390/ph17070938 (PMC11279418; doi:10.3390/ph17070938)
Supplement: Supplementary file 1 [file pharmaceuticals-17-00938-s001.zip › supplementary materials.pdf]

Supplementary materials

Supplementary Table S2: Primers used for the construction of the transporter libraries.

Transporter	Fw primer sequence	Rv primer sequence
Vector	CCTTAATTAAACCTCAGCGCCTTAATTAATGTTTTGCCTCAGCCTCATCCGCTCTAACCGaaaagg	ATGCACGCGUTCGCACGCATTCCGTTGG
pTef1	ACGCGTGCAUGCACACACCATAGCTTC	CCTTAATTAATGTTTTGCCTCAGCCCCGGGTTTTTtgtaattaaaacttagattagattgc
yEGFP	GGCTTAAUATGTCTAAAGGTGAAGAATTATTC	GGTTTAAUTCATTTGTACAATTCATCCATAC
YAL022C	GGCTTAAUATGAGTACTAGTGCGGACACTG	GGTTTAAUTCACCTGATAATAAAGTCAATTATG
YBL020W	GGCTTAAUATGGCGAAAAAAAACTCACAATTG	GGTTTAAUTCATACATCTTTAGAATTGGAAACCG
YBR147W	GGCTTAAUATGAAGCTGATCCCAATTATTTTG	GGTTTAAUTCAATTATCTATTAGTATTTTCTCG
YBR235W	GGCTTAAUATGGTTAGTAGGTTTTATCAG	GGTTTAAUTCATAATGCAGTAGTTACTGTC
YBR287W	GGCTTAAUATGGTTGAAACATTTAGTTTTGC	GGTTTAAUTCAAGTTGGGTTAGCCCACTG
YCL002C	GGCTTAAUATGCTTGTTATTGTTCTGCAGGGC	GGTTTAAUTCATAGTTTTCCTTTTGGCAACCGTGG
YCR075C	GGCTTAAUATGGTGTCGTTAGACGATATAC	GGTTTAAUTCACAGCGGGTACTCTGACGC
YEL004W	GGCTTAAUATGTGGAACTCACTAAAAGCATTC	GGTTTAAUTCATTTACTTTTCTTTATCGCCCC

YER039C	GGCTTAAUATGATTTATACGTCGTCAAAGAGC	GGTTTAAUTCACTTCTCAAGAGTAGCTGCTAG
YHR032W	GGCTTAAUATGTCTAAACAATTTAGTCATACC	GGTTTAAUTCAGTTATACCCAACCATAAGCC
YDL231C	GGCTTAAUATGAAAAGATATGAGCGAGATCG	GGTTTAAUTCAAAAGTTTTCTTCGCCCAGTATATC
YDR338C	GGCTTAAUATGGCTGGAATTTTGTCAAAGAC	GGTTTAAUTCAAGCCCCTAAGAGGGCTGTG
YDR352W	GGCTTAAUATGTCGTGCTCAAACGGCATCTGG	GGTTTAAUTCATGTTTGCGTTTCACCAGACAAC
YDR438W	GGCTTAAUATGAATCGTGTTGGTATAGACG	GGTTTAAUTCAGGCATCCAAGATAGGTCC
YGL084C	GGCTTAAUATGTCGCTGATCAGCATCCTGTC	GGTTTAAUTCAGCATTTTAGGTAAATTCCGTGC
YGL140C	GGCTTAAUATGTCGCTTAAATCAAAGTTGAC	GGTTTAAUTCAGTCCTTCTTTTCTTTCATGTG
YJL193W	GGCTTAAUATGTTTCAACAGCTGTCGGC	GGTTTAAUTCACTCAGGACGTATTTTCGC
YLR152C	GGCTTAAUATGTCCCTTTCTCTGGGTGCCGC	GGTTTAAUTCAAACTTTTAAATCGACCTTTAAGG
YML018C	GGCTTAAUATGGTGTCGAAGGATCAAACGTCC	GGTTTAAUTCAGTTGTTTGCTGCTGGCACCTC
YML038C	GGCTTAAUATGAATAGGACTGTCTTTTTGGC	GGTTTAAUTCAGACCTGCTGGGATGAACGAC
YMR034C	GGCTTAAUATGAAGACTCAGTACTCTCTAATA	GGTTTAAUTCACCTTGGTTGTGTATATGGGAC
YMR253C	GGCTTAAUATGAATCCATCAGTACCGAAGG	GGTTTAAUTCATTTAGAATCGGATAGGTCAAAC
YNL095C	GGCTTAAUATGGTGCACATTACTCTGGGTC	GGTTTAAUTCAAAGGTTCATCTGTACTTTCAG
YOL060C	GGCTTAAUATGTCGTTTTTGCCACTAAGGTC	GGTTTAAUTCATCTTTTTATGGTCGACGAGGAG

YOL092W	GGCTTAAUATGCAACTTGTGCCGCTAG	GGTTTAAUTCAATTCAGTATAAATTTTTTATTTC
YOR092W	GGCTTAAUATGACACACATCACACTGGGAC	GGTTTAAUTCATAATTTCATTTGAACTTTGATG
YOR271C	GGCTTAAUATGGCATCATCAGTCCCAGGGC	GGTTTAAUTCAAATACCTCTGTTAAAATAGACC
YOR307C	GGCTTAAUATGATTCAAACGCAAAGTACAGCG	GGTTTAAUTCATGCCTGACGTCCATCTTTTTTGG
YPL189W	GGCTTAAUATGTCGATGTTAAGAATCTGGAG	GGTTTAAUTCAACATTTCAAGTTGATGCCATG
YPL244C	GGCTTAAUATGGCGGGAAGTACATCCAG	GGTTTAAUTCACGCAGATTTTGCCTTCGG
YPL264C	GGCTTAAUATGACGCTGCAAAGAATTAGTAAA	GGTTTAAUTCAATCCTCCAAATCATCTAATTC
YPR201W	GGCTTAAUATGTCAGAAGATCAAAAAAGTG	GGTTTAAUTCAATTTCTATTGTTCCATATATAATA
YCR011C		
YHL035C		
YIL013C		
YKL188C		
YKL209C		
YKR103W		

YKR104W		
YDR011W		
YDR135C		
YDR406W		
YGR281W		
YLL015W		
YLL048C		
YLR188W		
YMR301C		
YNR070W		
YOL075C		
YOR011W		

YOR153W		
YOR328W		
YPL058C		
YPL147W		
YPL270W		
Jen1-3xHA		
Jen1-Myc		
Snq2-3xHA		

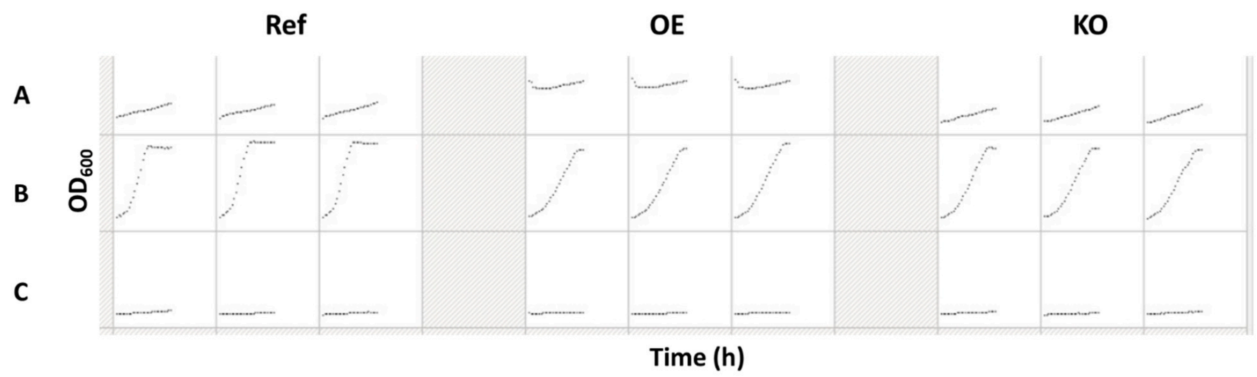

Supplementary Figure S1: Growth profiles of triplicate cultures of the reference strain BY4741 (Ref), the overexpressing strain (OE) and the knockout strain (KO) under different conditions. Growth of original strains on glycerol as carbon source (A); were compared to mitochondrially deficient (Rho$^-$, ρ^-) versions of each strain, using glucose (B) and glycerol (C) as carbon sources.

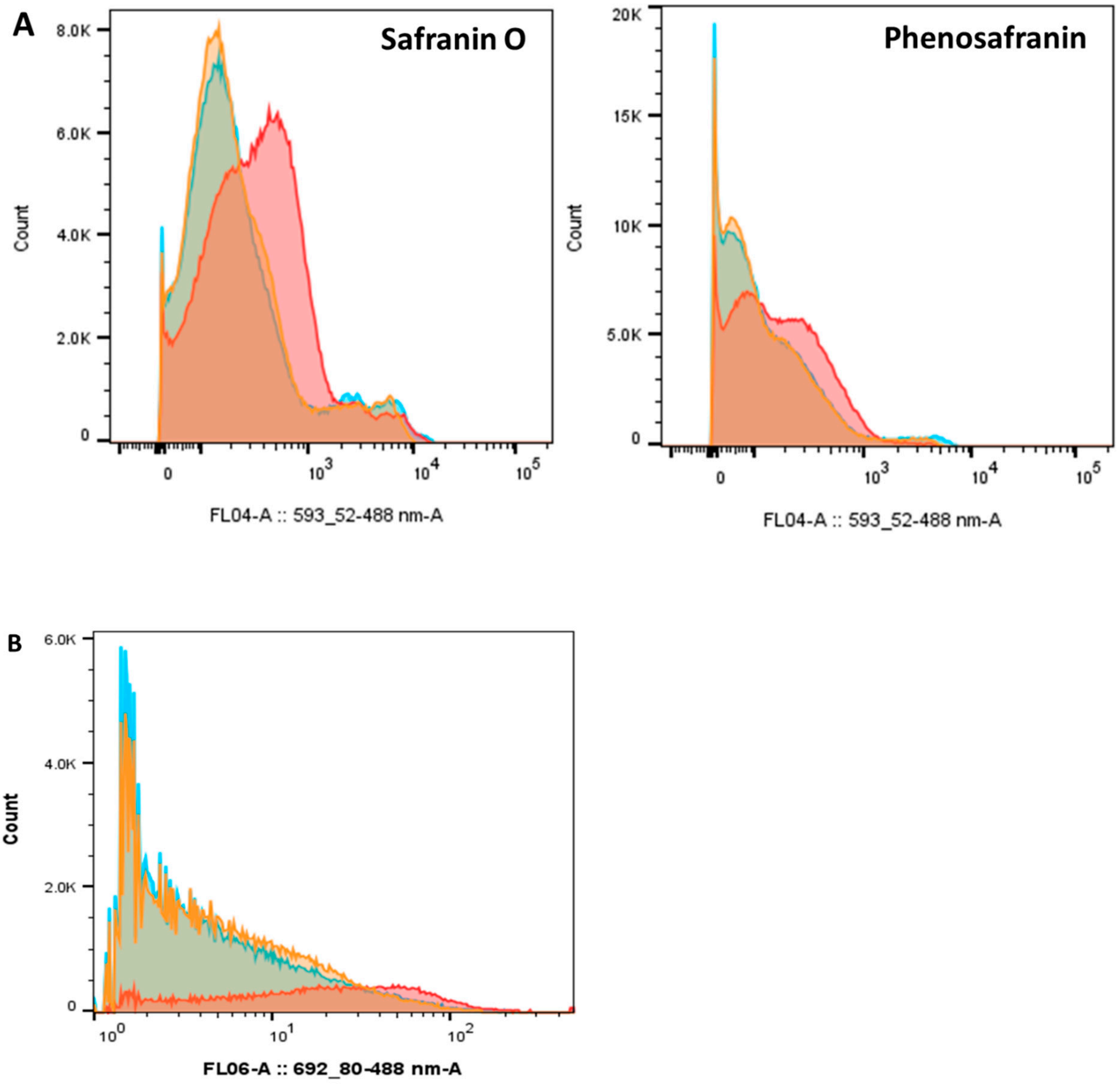

Supplementary Figure S2: Differential uptake of (A) safranin O vs Phenosafranin; and (B) bilirubin, by the reference strain BY4741 (orange), the strain overexpressing YLR188W (red) and the YLR188W-knockout strain (blue).

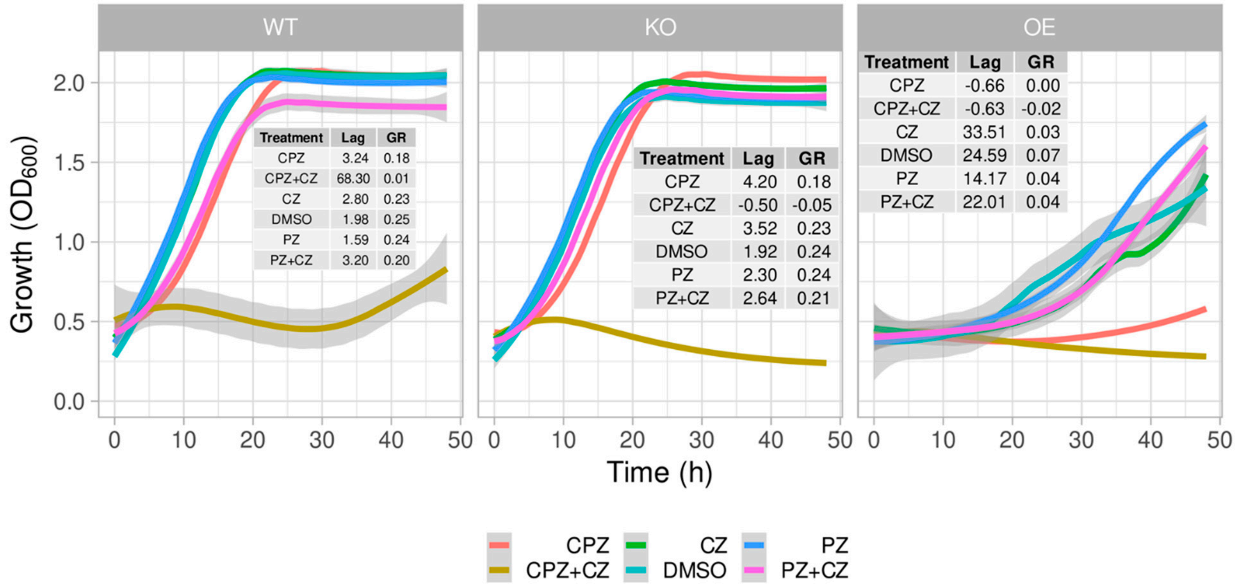

Supplementary Figure S3: Effects of different drugs and drug combinations on the growth of the reference strain BY4741 (WT), the strain overexpressing YLR188W (OE) and the YLR188W-knockout strain (KO). CPZ – chlorpromazine, CZ – clozapine, DMSO – dimethyl sulfoxide, PZ – prazosin.
